# Site-specific fluorescence dynamics in an RNA ‘thermometer’ reveals the role of ribosome binding in its temperature-sensitive switch function

**DOI:** 10.1093/nar/gku1264

**Published:** 2014-12-03

**Authors:** Satya Narayan, Mamta H. Kombrabail, Sudipta Das, Himanshu Singh, Kandala V. R. Chary, Basuthkar J. Rao, Guruswamy Krishnamoorthy

**Affiliations:** 1 Department of Chemical Sciences, Tata Institute of Fundamental Research, Mumbai 400005, India; 2 Department of Chemistry, Indian Institute of Technology, Kanpur 208016, India; 3 Tata Institute of Fundamental Research, Center for Interdisciplinary Sciences, Hyderabad 500075, India; 4 Department of Biological Sciences, Tata Institute of Fundamental Research, Mumbai 400005, India

## Abstract

RNA thermometers control the translation of several heat shock and virulence genes by their temperature-sensitive structural transitions. Changes in the structure and dynamics of MiniROSE RNA, which regulates translation in the temperature range of 20–45°C, were studied by site specifically replacing seven adenine residues with the fluorescent analog, 2-aminopurine (2-AP), one at a time. Dynamic fluorescence observables of 2-AP-labeled RNAs were compared in their free versus ribosome-bound states for the first time. Noticeably, position dependence of fluorescence observables, which was prominent at 20°C, was persistent even at 45ºC, suggesting the persistence of structural integrity up to 45ºC. Interestingly, position-dependent dispersion of fluorescence lifetime and quenching constant at 45°C was ablated in ribosome-bound state, when compared to those at 20°C, underscoring loss of structural integrity at 45°C, in ribosome-bound RNA. Significant increase in the value of mean lifetime for 2-AP corresponding to Shine–Dalgarno sequences, when the temperature was raised from 20 to 45°C, to values seen in the presence of urea at 45°C was a strong indicator of melting of the 3D structure of MiniROSE RNA at 45°C, only when it was ribosome bound. Taken all together, we propose a model where we invoke that ribosome binding of the RNA thermometer critically regulates temperature sensing functions in MiniROSE RNA.

## INTRODUCTION

Although the information content of an RNA primarily resides in its sequence, the conformation and dynamics of sub-regions of mRNA are emerging as critical regulators of expression of these messages encoded in the sequence (1–7). These regulatory regions, which are called riboswitches, are 5′-untranslated (UTRs) segments of RNA, which modulate the level of translation of RNA depending on changes in cellular environment. They operate in a variety of sensory modes by binding to metabolites or small molecules (1–6), or with a small change in temperature (3). Recently, there has been a report on how ligand binding and temperature sensing events are coupled (8).

Sensing and regulation of temperature is of vital importance in living cells. In bacteria, expression of heat-shock and cold-shock genes is regulated by temperature (9–12). Expression of virulence genes in pathogenic bacteria is also controlled by temperature (13,14). Short sequences of RNA in the UTR have been identified as thermo-sensing riboswitches in several systems perforcing them to be referred to as ‘RNA thermometers’ (9–13). In general, such ‘RNA thermometer’ sequences were shown to undergo temperature-induced conformational changes (9,10,15,16). The ROSE (Repression Of heat Shock gene Expression) element of mRNA present in the 5′-UTR of small heat-shock genes in many Gram-negative bacteria is known to function as an RNA thermometer by controlling translation in the temperature range of 30–45°C by blocking the initiation of translation till 30°C and allowing it at ∼40°C and beyond (9–12). The Shine–Dalgarno sequence (-GGAGGA-) strand (abbreviated hereafter to SD), which is a part of the RNA thermometer sequence, has been proposed to be inaccessible to ribosome binding due to its paired structure at low temperatures (<30°C) (9,10,15,16). The same is proposed to unfold at elevated temperatures (>40°C) due to the RNA's temperature sensing (RNA thermometer) property leading to the exposure of the SD sequence strand for ribosome binding, which in turn triggers the initiation of protein translation. Nuclear magnetic resonance (NMR) studies on a variant of the ROSE sequence showed a model that the structured RNA segment opens up at 45°C, thus facilitating the onset of SD sequence binding to ribosome (9). However, the question as to whether the hairpin-loop-structured ROSE segment opens up completely on its own or undergoes temperature-induced enhancement in dynamics during or following ribosome binding has not been addressed. The role of ribosome binding itself on the conformational dynamics of RNA thermometer sensors is unexplored. This issue becomes pertinent in view of the role of RNA dynamics in modulating the overall function as well as the newly found role of low-abundant transient structural states of RNA control function by responding to cellular cues (6,17,18).

In the present study, we explore temperature-dependent structure and dynamics of ‘MiniROSE’ RNA, a smaller segment of the thermometer sequence (9) by using 2-aminopurine (2-AP; a fluorescent analog of adenine) located at various sites of the MiniROSE RNA sequence. In general, the fluorophore 2-AP has found effective usage in bringing out several mechanistic details associated with the function of nucleic acids (19–23). The 2-AP pairs with U and the stability of such a base pair is analogous to that of A-U base pair (24–26). The fluorescence of 2-AP is highly sensitive and it is a site-specific reporter of structure and dynamics through observables such as its excited state lifetime and rotational diffusion kinetics. Base stacking with adjacent bases, strength of base pairing, extra-helical flipping out of bases, etc., have been quantitated using 2-AP observables stated above (19–23,25–27). The results from the present study point toward a novel mechanism of action for the RNA thermometer, whereby temperature-induced enhancement in the base-pair dynamics of the MiniROSE RNA leads to the opening of the SD segment of the hairpin thermometer RNA, specifically when it is ribosome bound, thus triggering the initiation of translation. This study thus, for the first time, underscores the importance of the ribosome complexes *per se* in modulating the structural transition and therefore the functional status of RNA thermometer, *in vitro*, a step closer to *in vivo* setting.

## MATERIALS AND METHODS

### Materials and characterization

**Figure 1.**
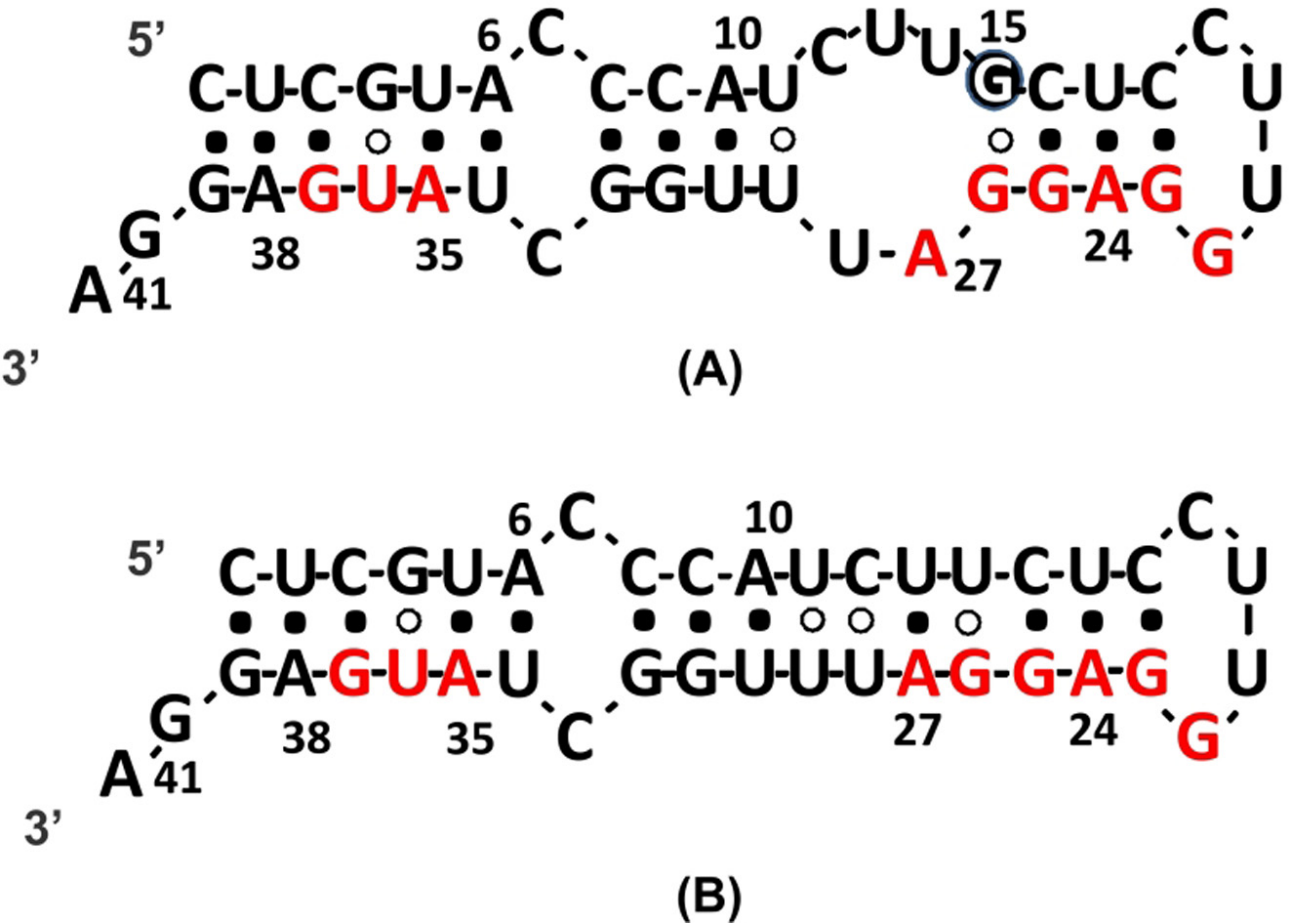
(**A**) Schematic representation of the wt MiniROSE RNA construct. The adenine residues replaced with 2-AP one at a time are shown in bold. The Shine–Dalgarno (SD) sequence and the AUG start codon are contained in the sequence. The conserved residue G15 is highlighted in a black circle. The Watson–Crick base pairs (G:C and A:U) are identified with filled circles and the non-canonical base pairs (G:U) are identified with open circles. (**B**) 15D MiniROSE RNA. Base-pairing scheme is based on the NMR-derived structure (9).

**Figure 2.**
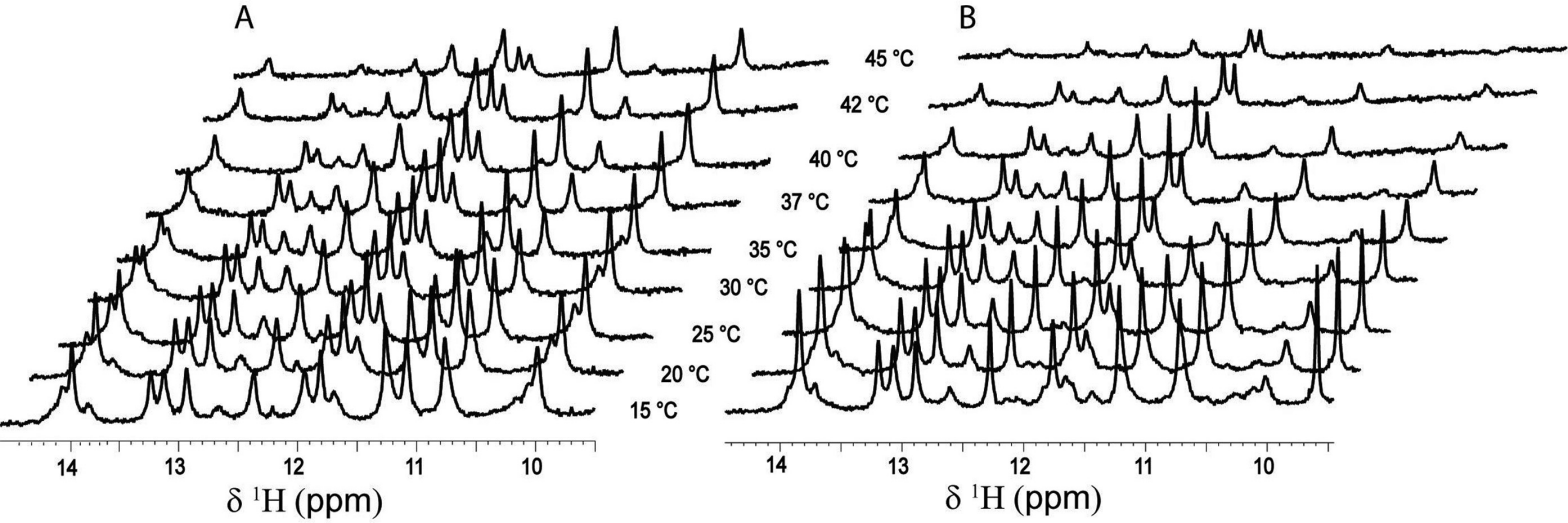
Temperature dependence of the imino-proton region of the 1D ^1^H NMR spectra of the wild-type (**A**) and the loop-mutant (CUUG changed to UUCG; (**B**)) of MiniROSE RNA in the temperature range of 288–318 K.

The MiniROSE RNA sequences containing 2-AP at seven different sequence positions (Figure 1) were purchased from the Fidelity Systems Inc. The RNAs were purified on denaturing polyacrylamide gels and desalted by the supplier. Their concentrations were quantified by measuring the absorbance at 260 nm and expressed as total nucleotide concentration. The buffer used in all the experiments was 10-mM Tris-HCl (pH 7.5), 5-mM MgCl_2_ and 150-mM KCl. Homogeneity of folded forms of RNA is essential for correct interpretation of fluorescence observables. 1D ^1^H NMR spectra (Figure 2) and native polyacrylamide gel electrophoresis (PAGE) profile (data not shown) of both the wild-type MiniROSE RNA and its loop-mutant (-CUUG- changed to -UUCG-; see the text later) samples showed that they are well folded and have homogeneous populations. Furthermore, dynamic light scattering measurements on both these RNAs showed that their hydrodynamic radii are identical (∼1.8 nm) suggesting that the RNA folds are similar where native PAGE analyses revealed their monomeric status. Acrylamide and urea were purchased from USB Corporation and used without further purification. Concentration of urea (stock solution) was ∼7 M as determined by refractive index measurement.

### Ribosome Extraction

A mixture of ribosome particles (30S, 50S and 70S) was purified from *Escherichia coli* cell-free extracts using Sephacryl S300 gel-filtrationchromatography (28–30). Sephacryl S300 gel-filtration cannot resolve the ribosomal sub-units and yields a mixture of ribosomal sub-units in the void-volume peak (30). *E. coli* cells were washed in buffer A [Buffer A: 10-mM Hepes-KOH (pH 7.5), 70-mM KCl, 10-mM MgCl_2_]. Cells (∼50-g wet-weight) were then resuspended in 100-ml polymix buffer [Polymix buffer: 20-mM Hepes-KOH (pH 7.5), 5-mM MgCl_2_, 0.5-mM CaCl_2_, 8-mM putrescine, 1-mM spermidine, 5-mM NH_4_Cl, 95-mM KCl, 1-mM dithioerythritol] containing 100-μg DNase. Cells were lysed by grinding the cell-paste with alumina. It was then centrifuged twice at 28 000 g for 30 min to clear cell debris, etc. Solid ammonium sulfate was added to the supernatant thus obtained, while stirring it to the final concentration of 210 mg/ml. The solution-pH was adjusted to 7.5 using ammonium hydroxide. Stirring was continued for another 30 min, followed by centrifugation at 28 000 g for 30 min. Clear yellow supernatant (crude ribosome preparation) was then loaded onto a column of Sephacryl S300 (GE Healthcare lifesciences) pre-equilibrated with buffer B (Buffer B: same as polymix buffer except NH_4_Cl is made 1 M). Ribosome fraction, a mixture of sub-units, eluted in the excluded volume, was primarily detectable by eluent turbidity. Absorbance was monitored at 260 nm. Peak fractions with majority of A_260_ material were pooled. Ribosome particles were precipitated by adding 100-mg PEG_6000_ per ml with stirring for 30 min at 0°C. The precipitated ribosomes were recovered by centrifuging at 16 000 g for 15 min. Pellets of ribosomes were redissolved in polymix buffer and methanol was added to the final concentration of 30% (v/v). Such final preparation of ribosome particles was dialyzed against polymix buffer containing 30% (v/v) methanol for 10 h. The absorbance of the preparation was measured at 260 and 280 nm and A_260_/A_280_ was found to be ∼1.8 and the final concentration of ribosome stock solution was 480 nM (Considering 1 A_260_/ml = 23 pmol/ml) (28). Samples were stored at –20°C in small aliquots. The quality of ribosome preparation was assessed by analyzing the ribosomal RNA constituents by agarose gel electrophoresis.

### RNA thermometer sample preparation

RNA samples taken in 10-mM Tris-HCl (pH 7.5), 5-mM MgCl_2_ and 150-mM KCl were heated to 90°C for 2 min, then quench-cooled using liquid nitrogen. RNA concentrations were found to be in the range of 6–15 μM of RNA molecule.

### Fluorescence measurements

#### Time-resolved fluorescence spectroscopy

Time-resolved fluorescence intensity and anisotropy decay of 2-AP was measured by employing a passively mode-locked frequency-doubled Nd:YAG laser (Vanguard, Spectra Physics) driven rhodamine 6G dye laser, which generates pulses of width 1 ps at 4 MHz (19–23). 2-AP in RNA was excited by using the second harmonic output (305 nm) of an angle-tuned Potassium dihydrogen phosphate (KDP) crystal. The excitation at 305 nm did not cause any cross-linking in the RNA, which was checked by urea-PAGE of the RNA sample before and after the experiments (data not shown). Fluorescence emission was collected through a polarizer and a 345-nm cut-off filter followed by a monochromator at 370 nm with a collection bandwidth of 3 nm. The cut-off filter was used to prevent scattering of the excitation beam from the sample. The number of counts in the peak channel was at least 10 000. We performed extensive checks to ensure that 10 000 counts at the peak are sufficient to recover the lifetime components reliably. Increasing the counting beyond 10 000 at the peak did not improve the quality of fits as slow time instability of laser and the detector contributed to systematic errors. Fluorescence decay curves were obtained by using a time-correlated single-photon counting setup, coupled to a micro-channel plate photomultiplier (model 2809U; Hamamatsu Corp). The instrument response function (IRF) was obtained at 305 nm using a dilute colloidal suspension of dried nondairy coffee whitener. The half-width of the IRF was ∼40 ps. In fluorescence lifetime measurements, the emission was monitored at the magic angle (54.7°) to eliminate the contribution from the decay of anisotropy. In time-resolved anisotropy measurements, the emission was collected at directions parallel (ll) and perpendicular (⊥) to the polarization of the excitation beam.

The experimentally obtained fluorescence decay traces, *I*(*t*), were analyzed by nonlinear least-square iterative deconvolution method based on the Levenberg–Marquardt algorithm (31) and expressed as a sum of exponentials with equation,

(1) \begin{equation*}
I(t) = \sum\nolimits_i {\alpha _i \exp ( - t/\tau _i )},
\end{equation*}

where *α_i_* is the amplitude of the *i*th component associated with fluorescence lifetime *τ_i_*, such that ∑*α_i_* = 1. The mean lifetime $\tau _m = \sum\nolimits_i {\alpha _i \tau _i }$ that is proportional to fluorescence quantum yield gives us information on the average fluorescence yield of the system.

The time-resolved fluorescence anisotropy decay curves were obtained by measuring the fluorescence emission intensity at parallel (*I*_∥_(*t*)) and perpendicular (*I*_⊥_(*t*)) directions to the polarization of the excitation beam and constructing the time dependent anisotropy, *r*(*t*), with the following equation:

(2) \begin{equation*}
r(t) = \frac{{I_{||} (t) - G(\lambda )I_ \bot (t)}}{{I_ \bot (t) + 2G(\lambda )I_ \bot (t)}},
\end{equation*}

where *G*(λ) is the grating factor at the wavelength λ of emission. The magnitude of *G*-factor for the emission collection optics was determined independently using a standard solution of 2-AP for which the fluorescence lifetime was 11.3 ns using standard procedures (19–23,32).

Time-resolved anisotropy decays were analyzed with the following sets of equations:

(3) \begin{equation*}
I_{||} (t) = \frac{1}{3}I(t)[1 + 2r(t)]
\end{equation*}

(4) \begin{equation*}
I_ \bot (t) = \frac{1}{3}I(t)[1 - r(t)]
\end{equation*}

(5) \begin{equation*}
r(t) = r_0 \left\{ {\beta _1 \exp ( - \tau /\phi _1 ) + \beta _2 \exp ( - \tau /\phi _2 )} \right\}
\end{equation*}

(6) \begin{equation*}
\phi _{\rm m} = \sum {\beta _{\rm i} \phi _{\rm i} },
\end{equation*}

where *r*_0_ is the initial anisotropy, *β*_i_ is the amplitude of the *i*th rotational correlation time (*ϕ_i_*) such that ∑*β_i_* = 1 and *ϕ*_m_ is mean rotation correlation time. The *ϕ*_m_, which is an operational parameter, takes note of both the local (*ϕ*_1_) and the global dynamics (*ϕ*_2_) and thus gives an indication of the overall rotational dynamics felt by 2-AP. The *ϕ*_m_ is proportional to the area under the anisotropy versus time curve. Furthermore, the *ϕ*_m_ is proportional to the steady-state anisotropy. Anisotropy decay parameters were obtained by deconvoluting the decays (Equations (3-5)) with IRF by keeping the initial anisotropy (*r*_0_ = 0.31 estimated in a separate experiment on 2-AP in 50% glycerol) and the intensity decay parameters (*α_i_* and *τ_i_*) estimated from Equation (1) fixed during the analysis. The goodness of the fits was checked by both the reduced χ^2^ value and randomness of residuals; for more details see (19–23).

The time resolution is limited largely by the width of the IRF, which is 40 ps. We have obtained, in several samples, fluorescence lifetimes and rotational correlation times in the range of 0.1–0.3 ns. Since these values are quite close to both the width of the IRF and the time/channel used, both of which are ∼40 ps, their reliability was checked. We measured the rotational correlation time (*ϕ*) of a small molecule *N*-acetyltryptophanamide in glycerol–water mixtures with viscosity (*η*) in the range 1–5 cP. The values of *ϕ* estimated were 0.060 ± 0.027, 0.121 ± 0.034, 0.224 ± 0.029 and 0.315 ± 0.029 ns, when the solvent viscosities were 1.0, 2.1, 4.4 and 5.4 cP, respectively, as expected from the Stokes–Einstein relationship (*Φ* = *ηV*/*kT*, where *V* is the molecular volume).

#### Fluorescence quenching

The solvent accessibility of 2-AP residues was monitored by collisional quenching with acrylamide. Titration stock was made with ∼5-M acrylamide in 10-mM Tris-HCl (pH 7.5), 150-mM KCl with 5-mM MgCl_2_ and the acrylamide was added to a final concentration range of 0–200 mM. Fluorescence lifetimes were measured immediately after mixing. Acrylamide quenching titrations were fitted to the Stern–Volmer equation (32) for analysis of collisional quenching:

(7) \begin{equation*}
\tau _0 /\tau = 1 + k_{\rm q} \tau _0 [Q],
\end{equation*}

where *τ*_o_ and *τ* are the lifetimes in the absence and presence of quencher, respectively, [*Q*] is the acrylamide concentration and *k*_q_ is the bimolecular quenching rate constant (M^−1^ s^−1^).

### Nuclear magnetic resonance

Temperature dependence of the imino-proton region of the 1D ^1^H NMR spectra of the wild-type and the loop-mutant of MiniROSE RNA was recorded in the range 288–318 K on a Bruker Avance 800-MHz NMR spectrometer equipped with a 5-mm cryogenically cooled triple-resonance probe and pulse-field gradients, with ^1^H carrier placed on H_2_O resonance (4.68 ppm) (33–35). The samples taken in 3-mm NMR tubes were of 0.6 mM in their RNA concentration. The data thus collected were processed with XWINNMR (Bruker) and analyzed with TOPSPIN-3.1. The spectra thus obtained at different temperatures are shown in the form of a stacked plot (Figure 2) for better visualization.

## RESULTS AND DISCUSSION

### Site-specific fluorescence dynamics in MiniROSE RNA

MiniROSE RNA is the functional domain of an RNA thermometer sequence (Figure 1). NMR structural studies on a variant MicroROSE, a shorter and engineered mutant (sequence altered at the loop) version, had shown this RNA sequence to be well structured (9). The focus of this study was largely on aspects of structural transition the natural version of RNA exhibits as a function of temperature (9). The conclusions drawn were therefore centered mostly on the structural transitions that lead to the opening-up of biologically relevant sites on the MiniROSE RNA, namely the SD sequence and the AUG start codon (Figure 1). So far, there has been no clear discussion on the dynamic transitions of this RNA in the presence of ribosome. In the current study, we employed 2-AP-based reporting of fluorescence dynamics of full-length MiniROSE RNA to uncover hitherto unexplored effects of ribosome on the RNA thermometer transitional dynamics. We chose all the seven adenine residues present in MiniROSE RNA for replacing them with 2-AP, one at a time (Figure 1), and investigated systematic changes in fluorescence observables in the absence and presence of ribosomes. The observables that could be measured are the following: (i) the fluorescence lifetime, which is a reliable indicator of the level of base stacking interaction of the 2-AP with the neighboring bases, the strength of base pairing (21,26) and a sensitive read-out of extra-helical popping of the base (27); (ii) fluorescence depolarization kinetics which quantifies, in a direct way, the rotational dynamics of 2-AP, both in its local or intrinsic mode and in the mode coupled to the nucleotide backbone (encompassing non-local motions) to which the 2-AP is attached (19–23,26,36,37). Information on both these modes of rotational dynamics provided insightful solutions to several important problems in DNA repair biology in recent years (19,20) and (iii) quenching of 2-AP by acrylamide. Bimolecular quenching constant, *k*_q_, estimated from dynamic quenching of fluorescence, has been of immense use in revealing the dynamic aspects, especially related to solvent accessibility of structures in biomolecules in general (38–40). Buried solvent accessible area of ligands in RNA aptamer complexes was shown earlier to correlate with their binding energy (41) and therefore expected to offer an important read-out in the MiniROSE RNA as well.

Thus, the seven RNA constructs were used to measure the above-mentioned dynamic fluorescence parameters and explore the temperature-dependent changes in the structure and dynamics vis-a-vis wild-type MiniROSE RNA thermometer in its free versus ribosome-bound states. The study was carried out under the following three conditions: (i) at 20ºC, a temperature at which translation is inhibited where the MiniROSE RNA strand is supposed to be well structured, thus concealing the SD sequence from ribosome recognition, appropriate for initiating translation; (ii) at 45ºC, a temperature at which translation is allowed, following structural transitions in MiniROSE RNA motifs; and (iii) at 45ºC in the presence of 7-M urea, an artificial condition under which all intramolecular (tertiary and secondary) interactions in the RNA are expected to be weakened, thereby essentially providing a control that has lost all plausible structures. This strategy was expected to offer us insights into the mechanism of temperature-sensitive translation through a comparison of structure and dynamics under the above-mentioned three conditions. Further, we compared the outcome of the first two conditions in free as well as ribosome-bound states of MiniROSE RNA.

### Site-specific fluorescence analyses of free (ribosome unbound) MiniROSE RNA

Fluorescence intensity decay kinetics of all the seven 2-AP-incorporated RNA samples showed the presence of four lifetime components as seen in previous studies in DNA (19–23). Free 2-AP has a fluorescence quantum yield of 0.68 and has a single fluorescence lifetime of 11.3 ns in aqueous solution (25,42). When 2-AP is incorporated into polynucleotides, its photophysics becomes complex when compared with that of its free state. This is evident from the observed multi-exponential decays with time constants ranging from 50 ps to ∼10 ns (19–23,25,26,42). Such heterogeneous lifetimes can be interpreted as due to distribution of partially stacked structures. The shortest lifetime component (∼50 ps) has been used to derive information on the level of duplex formation and the longest component (8–10 ns) is ascribed to conformations wherein the 2-AP is either extra-helical or unstacked. It is relevant to point out here that such interpretations of lifetimes vis-à-vis changes in base-pair properties have provided insightful transitions in several biological systems (19–23,26). The complex population heterogeneity observed through fluorescence lifetimes is not seen with techniques such as NMR, CD, etc. due to time averaging of various conformers during their relatively slower timescales of these techniques. The rapid timescale(s) (ps–ns) associated with fluorescence decay kinetics enables the visualization of these conformers as snapshot pictures.

**Figure 3.**
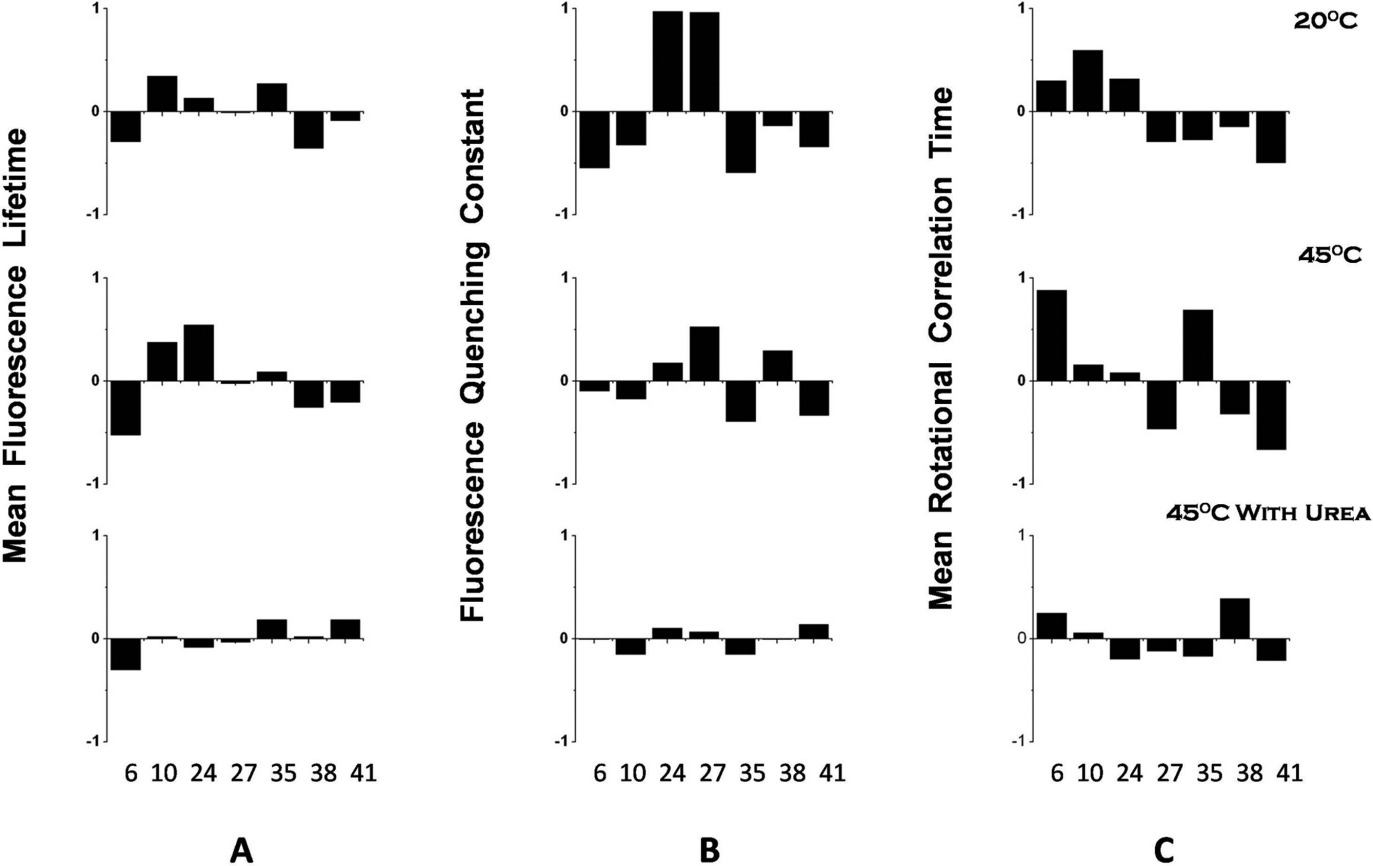
Site-specific variation of fluorescence parameters measured in the absence of ribosome. Variations are represented as (Observed value – Mean value)/Mean value. The Mean value is the mean of all the observed values at the seven locations. The three columns show (**A**) mean fluorescence lifetime (*τ*_m_,), (**B**) dynamic fluorescence quenching constant (*k*_q_) and (**C**) mean rotational correlation time (*ϕ*_m_), respectively. The three rows correspond to measurements at 20 , 45 and 45°C in the presence of 7-M urea respectively. The numbers along the X-axis refer to the position of 2-AP from the 5′ end of the MiniROSE RNA.

Table 1 shows the parameters obtained from fluorescence decay kinetics of the seven MiniROSE RNAs under the three conditions mentioned above. Only the mean fluorescence lifetime, *τ*_m_ ( = Σ*α_i_τ_i_*), is given in Table 1 as we address mainly changes in *τ*_m_ in this work. Individual lifetimes and their amplitudes are given in Supplementary Table S1. *τ*_m_ showed significant dependence on the position of 2-AP in MiniROSE RNA at 20ºC as expected for a structured RNA. Figure 3 shows a graphical representation of the position dependence of *τ*_m_ plotted as the variation from the mean value of the parameter averaged over all the seven positions. This representation brings out the position dependence in a stark manner. It is instructive to focus our attention on the site-specific variation of the fluorescence observables. As mentioned above, the individual lifetime components (Supplementary Table S1) could have their origin on the various sub-states of the 2-APconformation. The dominant factor that controls the lifetime of 2-AP in polynucleotides is the strength of stacking interactions of 2-AP with the neighboring bases leading to electron delocalization (25). Stronger stacking interaction leads to a lifetime component shorter than 100 ps resulting in a shorter value of the mean lifetime, *τ*_m_. The strength of stacking interaction reflects structural aspects such as base-pair interaction (19–23,25,26). It is worth mentioning here that the fluorescence lifetime of 2-AP in polynucleotides is also controlled by motional dynamics of 2-AP, which would be position dependent, especially near an open end (19).

**Table 1.** Parameters associated with decay of fluorescence intensity, fluorescence anisotropy and bimolecular quenching constant (*k*_q_) of 2-AP in MiniROSE RNA

2-AP-site^a^	Mean fluorescence lifetime, *τ*_m_ (ns)			Mean rotational correlation time, *ϕ*_m_ (ns)			*k*_q_^b^ (10^9^ M^−1^s^−1^)		
	20°C	45°C	45°C + urea	20°C	45°C	45°C + urea	20°C	45°C	45°C + urea
6	1.06 ± 0.05	0.60 ± 0.06	1.30 ± 0.04	2.82 ± 0.10	1.41 ± 0.09	0.98 ± 0.03	4.7 ± 0.3	7.5 ± 0.4	2.7 ± 0.2
10	1.86 ± 0.05	1.88 ± 0.04	1.65 ± 0.05	3.46 ± 0.08	0.87 ± 0.03	0.83 ± 0.05	7.0 ± 0.4	6.8 ± 0.3	2.3 ± 0.1
24	1.69 ± 0.08	2.12 ± 0.01	1.67 ± 0.03	2.86 ± 0.21	0.81 ± 0.03	0.63 ± 0.03	20.4 ± 0.9	9.7 ± 0.2	3.0 ± 0.2
27	1.34 ± 0.06	1.32 ± 0.02	1.72 ± 0.06	1.54 ± 0.05	0.40 ± 0.02	0.69 ± 0.04	20.3 ± 0.5	12.6 ± 0.4	2.9 ± 0.2
35	1.82 ± 0.01	1.49 ± 0.02	2.22 ± 0.07	1.58 ± 0.08	1.27 ± 0.05	0.65 ± 0.02	4.2 ± 0.2	5.0 ± 0.3	2.3 ± 0.1
38	0.91 ± 0.05	1.01 ± 0.03	1.90 ± 0.03	1.85 ± 0.11	0.51 ± 0.02	1.09 ± 0.03	8.9 ± 0.4	10.7 ± 0.5	2.7 ± 0.2
41	1.22 ± 0.05	1.05 ± 0.09	2.22 ± 0.05	1.09 ± 0.10	0.25 ± 0.02	0.62 ± 0.04	6.8 ± 0.3	5.5 ± 0.4	3.1 ± 0.1

^a^The number refers to the position of 2-AP in MiniROSE RNA from the 5′-end.

^b^*k*_q_ is the bimolecular quenching constant determined using the Stern–Volmer equation (Equation (7)).

The bimolecular quenching constant, *k*_q_, is a reliable indicator of solvent accessibility (38). The *k*_q_ values show large-scale variation among the various positions at 20°C (Figure 3). The absence of obvious correlation of *k*_q_ with the structural aspect of various positions is indeed perplexing although positions 24 and 27, which have higher values of *k*_q_ when compared to that of the other positions, are part of the SD sequence involved in binding to ribosome (9).

Local (*ϕ*_1_) and global (*ϕ*_2_) motional dynamics (see Equations (5) and (6)) of 2-AP derived from fluorescence anisotropy decay kinetics are listed in Supplementary Table S2. The mean rotational correlation time *ϕ*_m_ is given in Table 1. Although there is no obvious structural correlation for rotational dynamics, the observed faster dynamics associated with position 41 (Table 1, Supplementary Table S2 and Figure 3), which is the last residue of the single-stranded end of the RNA, is as expected. We prefer to be conservative in interpreting the signature patterns of these three observables vis-à-vis the 2-AP positions at a given condition, but wish to emphasize that the relative changes incurred while going from one set of conditions to another (e.g. 20°C versus 45°C versus the same in the presence of ribosomes, etc.) are more meaningful in deciphering the structural transitions of MiniROSE RNA, as described below.

Noticeably, the position dependence of fluorescence observables which was prominent at 20°C was largely persistent at 45ºC also (Table 1, Supplementary Tables S1 and S2 and Figure 3), suggesting persistence of structural integrity even at 45°C. It should be mentioned here that changes in the fluorescence parameters with change in temperature can arise due to the following two reasons: (i) Intrinsic temperature dependence caused by change in the non-radiative rate and change in the solvent properties and (ii) temperature change induced conformational changes in the RNA. While we expect no site specificity due to the first reason, the second source is expected to result in site-specific conformational changes. The observed persistence of structural integrity at 45ºC (inferred from site-specific conformational changes) was most surprising in view of the conclusion from the earlier work (9) which emphasized that the RNA loses its hairpin structure at 45ºC. It is the loss of hairpin fold at higher temperature (i.e. 45ºC) that has been described as a ‘thermo-sensor’ needed for the structural transition leading to accessibility of SD for ribosome binding (9). Although the profile of all the three fluorescence observables across the length of the MiniROSE RNA is not identical at 20°C versus 45°C, there is a gross similarity in the pattern at both the temperatures. Intriguingly, there were no uniquely discernible changes at 24 and 27 positions on MiniROSE RNA, the sites encompassing SD sequence, where structural transitions have been shown to be most acute (9).

On the other hand, the imino-proton region of the 1D ^1^H NMR spectrum (43–45) (Figure 2) showed all the expected exchangeable imino-proton resonances arising from G(1H) and U(3H) at 25°C, confirming that the wild-type MiniROSE RNA indeed adopts a hairpin conformation as shown in Figure 1. Further, the temperature dependence study of these imino-proton signatures revealed that they expectedly start broadening out with increase in temperature. However, observation of 10 imino-proton resonances, arising from both G and U residues even at as high as 45°C, reveal that the 3D structure of the RNA is still intact at this high temperature. Thus, NMR experiments revealed the persistence of the structural integrity for the wild-type MiniROSE RNA even at 45°C (Figure 2A). Similar observations could be made in the case of the loop-mutant of MiniROSE RNA (CUUG changed to UUCG) at lower temperatures (25–30°C). However, the spectral signatures of the imino-protons start broadening out at a much faster rate as compared to that of the wild-type RNA indicating its relative instability at higher temperatures, as expected. At 45°C, it is clear that the loop-mutant is mostly unfolded as we observed very few and faint imino-proton resonances (Figure 2B).

Rotational correlation times (both the short and the long correlation times), in general, were shorter at 45°C when compared to 20°C (Table 1 and Supplementary Table S2) indicting that the RNA was more dynamic at 45°C. The shorter correlation time arises from the local motion of 2-AP within the RNA and the longer correlation time represents a combination of segmental and global tumbling dynamics of the RNA similar to the observation in other analogous systems (19–23). Global tumbling of a compact folded structure of 41 nucleotide long RNA is expected to give a correlation time of ∼8 ns as observed in some of the locations (Supplementary Table S2). It is the general enhanced motional dynamics observed at 45°C that could be critical in releasing the SD sequence to bind productively to ribosome. Therefore, interaction of MiniROSE RNA with ribosome at this condition could be a crucial ‘driver’ for the structural transition that facilitates the translational onset.

Most strikingly, the profiles of all the observables were nearly flat in the presence of 7-M urea at 45°C (Table 1 and Figure 3). The changes in all the fluorescence observables saturated well before reaching the level of 7-M urea (data not shown). This effect of ‘complete erasure’ of the observed profile at 7-M urea at 45°C was expected, but served as a good control to artificially ablate all the structure in MiniROSE RNA. This indicated the absence of secondary structure in the RNA in the denatured state and also that the photophysics and dynamics of 2-AP was largely independent of the nucleotide sequence. The residual level of ‘non-flatness’ of the fluorescence observables in samples with urea at 45°C (Figure 3) could be the result of minor influence of near neighbor on the photophysics and dynamics of 2-AP and not due to residual structure of the RNA. It is well known that a base is subject to significant sequence-context effects even in the absence of the structure in nucleic acids (46). Lower value of *k*_q_ seen under this condition compared to the values in the absence of 7-M urea (Table 1) may seem counter-intuitive. However, it is interesting to note that bases in an unstructured single-stranded DNA are less solvent exposed (lower value of *k*_q_) when compared to the structured double-stranded DNA (21). Similar behavior is expected for RNA. This is most likely due to the tendency of unstructured nucleotides to collapse into a globule. It has been reported that the persistence length of single-stranded RNA is 0.8 nm as compared to 15 nm for base-paired structures (47).

The control set with urea at 45°C provided a contrast with the high-temperature set (45°C), where significant level of structural signatures (non-flat nature of observables) was still evident at 45°C, although there were indications of faster dynamics observed from the rotational correlation times (Table 1 and Supplementary Table S2). All the above-described results set the stage for a comparison of the same in the presence of ribosome binding to MiniROSE RNA, an important contrast for uncovering the effects related to protein translation control. Although the experiment below involving ribosome addition did not contain active components of translation, it takes the system one step closer to translation by providing the initial binding steps of MiniROSE RNA with ribosomes. The steps of translation onset being only downstream to proper recognition of SD by ribosome sub-units, one can uncouple translation from the recognition step, which is the focus of the current study.

### Site-specific fluorescence analyses of ribosome-bound MiniROSE RNA

The question we addressed here was whether the temperature dependence of translation is the result of temperature-dependent binding of MiniROSE RNA to ribosome or, at least partially, due to temperature-dependent structural transition of ribosome-bound MiniROSE RNA? More importantly, does the binding of MiniROSE to ribosome at low (20°C) versus high temperature (45°C) lead to any biologically relevant structural changes in ribosome-bound MiniROSE RNA as compared to the same in free MiniROSE RNA? This is a biologically relevant mechanistic question that has not received sufficient attention so far. Most high-resolution structural studies involving NMR have been limited to free RNA targets, devoid of ribosomes, due largely to NMR methodology constraints. Time-domain fluorescence observables being monitored in the current study are sensitive indicators of the impact of ribosome cues on MiniROSE RNA transitions, if there are any. To address this issue, binding of the MiniROSE RNA with ribosome was assessed at 20 and 45°C by monitoring time-resolved anisotropy decay kinetics of 2-AP. The ribosome preparation used is a mixture of sub-units collected from Sephacryl S300 gel-filtration void volume peak. The admixture of ribosome particles is expected to support initial binding of RNA by 30S particles followed by 50S sub-units required for SD opening in RNA.

**Figure 4.**
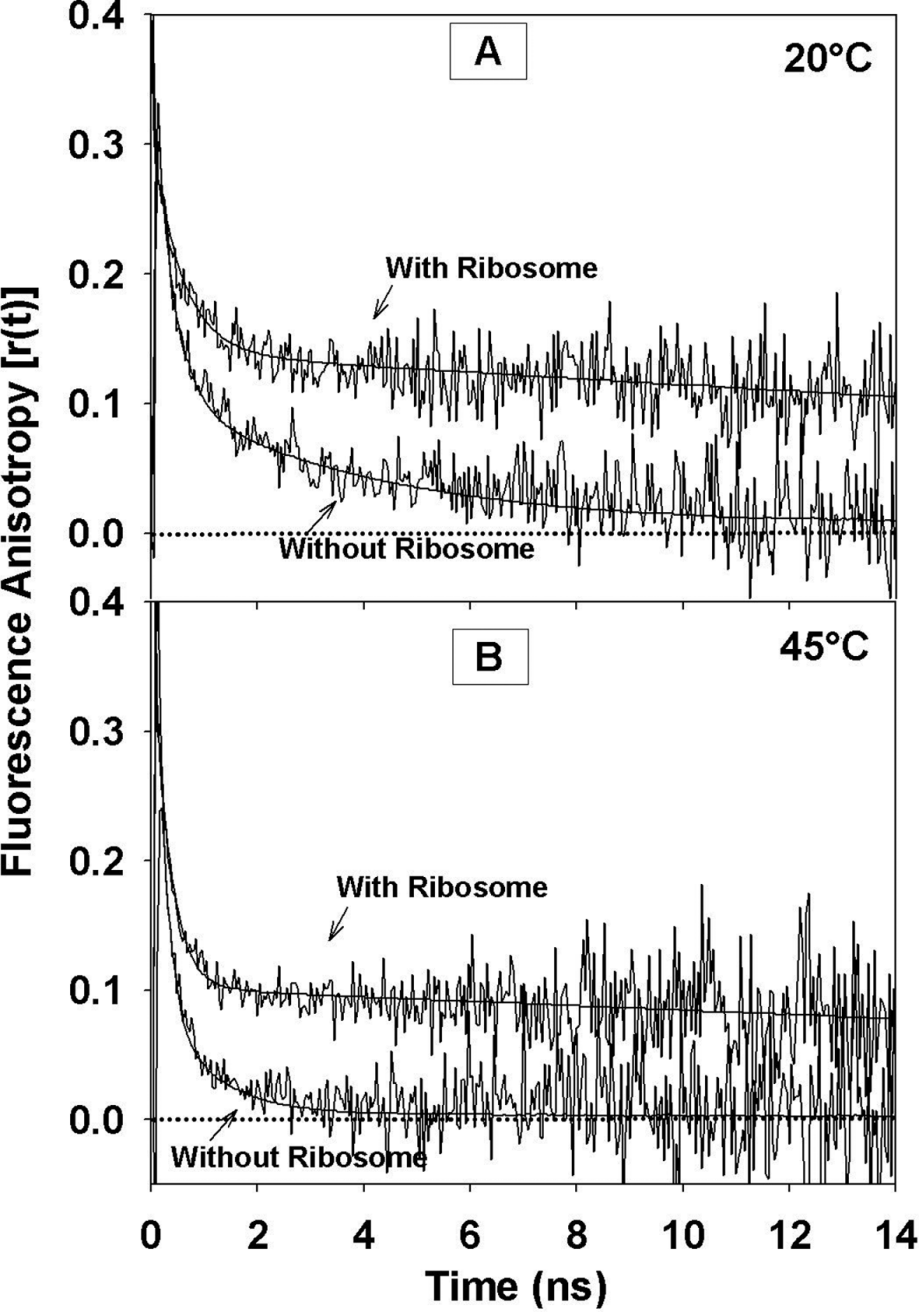
Decay curves of fluorescence anisotropy of MiniROSE RNA (2-AP at position ‘24’) in the absence and presence of ribosome at 20°C (panel (**A**)) and 45°C (panel (**B**)). Ribosome binding of MiniROSE RNA results in the observation of very long rotational correlation time (>50 ns) at both the temperatures.

**Figure 5.**
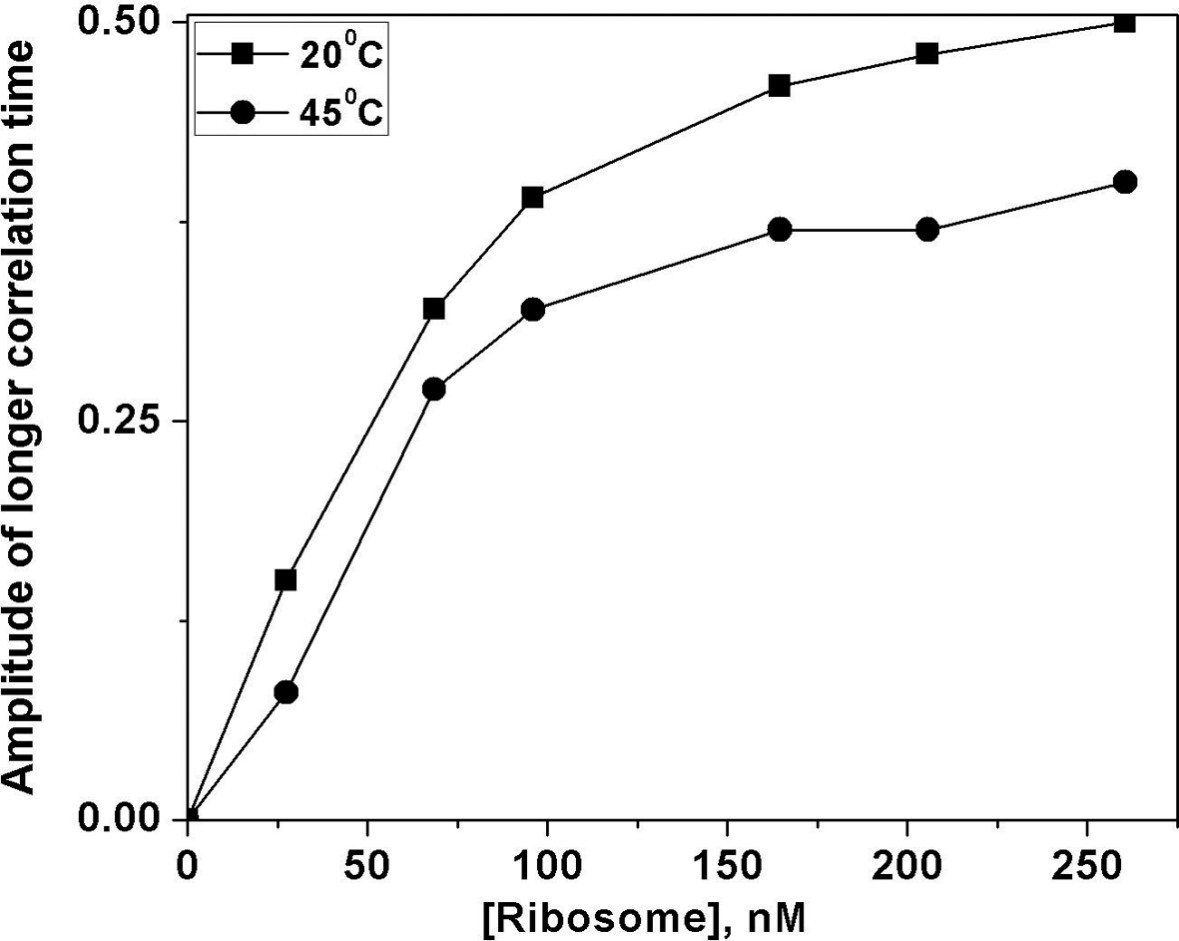
Dependence of the amplitude (*β*_2_) corresponding to the very long (>50 ns) correlation time on the concentration of ribosome at 20 and 45°C.

Binding to ribosome sub-units results in slowing down of the global tumbling dynamics of the RNA that could be observed as a rotational correlation time longer than the time window of our observation (Figure 4). Emergence of this long (>50 ns) rotational correlation time is a direct pointer to binding of RNA to ribosome. The amplitude associated with this correlation time increases with the increase in the extent of binding and hence it is a reliable indicator to derive the binding isotherm of the system (Figure 5). Based on the observed isotherm, we chose the minimum saturating level of ribosome to add such that all the RNA is in the bound form. Interestingly, MiniROSE RNA binding to ribosome was similarly robust at both temperatures (20 and 45°C). Lower temperature (20°C) did not hamper binding, perhaps due to high stability of ribosome sub-units even under these conditions. This result appears in contrast with the toe-printing studies, which showed temperature-dependent binding of similar RNA thermometer segments, such as the *agsA* mRNA from *Salmonella* (12) and RNA from *Pseudomonas putida and Pseudomonas aeruginosa* (48), to 30S ribosome. The ribosome preparation in the current experiments does not involve purified individual sub-units of ribosomes of the sort used for toe-printing studies. A dynamic mixture of sub-units present in our ribosome preparation can capture physiological milieu better. In this context, therefore temperature-independent binding of MiniROSE RNA is not totally surprising as such mixture of sub-units can offer binding surfaces that are temperature non-specific. In any case, we do not know the exact origin of this difference at the moment, although these results pertain to RNA sequences, which are slightly different (especially the bases flanking the SD sequence) from the MiniROSE RNA. As a word of caution, we want to state that it is likely that binding to ribosome at both 20 and 45°C may not be universal for all the similar thermometer sequences. Uncovering finer mechanistic details does require additional work involving reconstitution of different stages of ribosomal complexes with MiniROSE RNA substrates.

**Figure 6.**
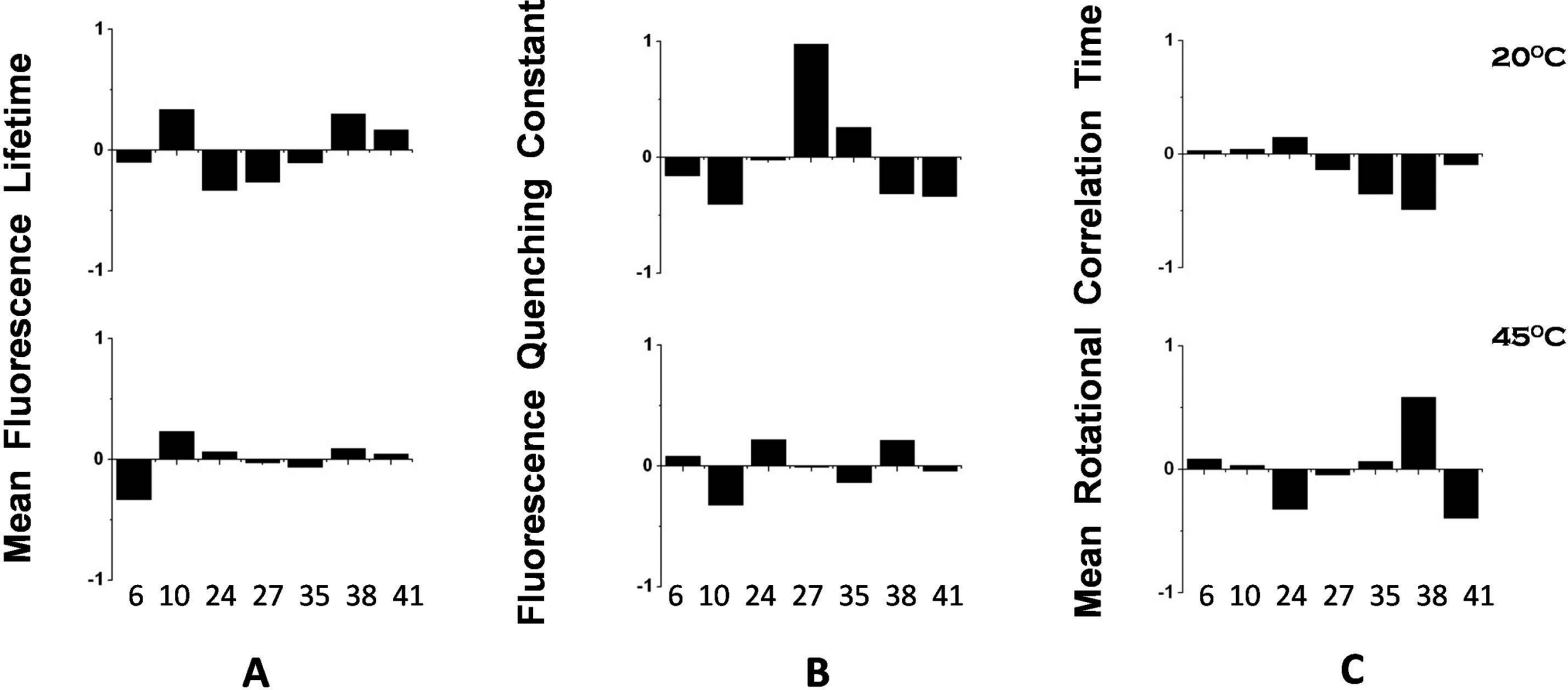
Site-specific variation of fluorescence parameters measured in the presence of ribosome. Variations are represented as (Observed value – Mean value)/Mean value. The Mean value is the mean of all the observed values at the seven locations. The three columns show (**A**) mean fluorescence lifetime (*τ*_m_), (**B**) dynamic fluorescence quenching constant (*k*_q_) and (**C**) mean rotational correlation time (*ϕ*_m_), respectively. The two rows correspond to measurements at 20 and 45°C, respectively. The numbers in the X-axis refer to the position of 2-AP from the 5′ end of the MiniROSE RNA.

Table 2 and Supplementary Table S2 show fluorescence parameters obtained for 2-AP-labeled MiniROSE RNA, when bound to ribosome at 20°C versus 45°C. Site-specific deviations from the mean value of the parameters are shown in Figure 6. Firstly, we note that the observed non-flat pattern of all the three parameters at 20°C is different from those obtained in the absence of ribosome (Figure 3). This in itself is not surprising since the binding is expected to modulate these parameters. However, the significant reduction in the level of position-dependent dispersion (non-flatness) of fluorescence lifetime (panel (A)) and the quenching constant (panel (B)) at 45°C when compared to those at 20°C is quite striking. This could be interpreted as due to the MiniROSE RNA retaining its structural integrity at 20°C even when it is bound to ribosome as compared to depletion of the same at 45°C, specifically in bound MiniROSE RNA. The persistence of position-dependent dispersion in the value of mean rotational correlation time at 45°C (panel (C)) may seem to argue against the above inference that the MiniROSE RNA is devoid of intra-RNA interactions when it is bound to ribosome at 45°C. The behavior of rotational correlation time (panel (C)) contrasts that of fluorescence lifetime (panel (A)) and the bimolecular quenching constant, *k*_q_ (panel (B)). This could be rationalized based on the origin of these three fluorescence-derived parameters, as reasoned below. Fluorescence lifetime, which is the result of photophysics of the excited state of 2-AP, is largely controlled by the interaction of the aminopurine with its near neighbors as a result of structure within the RNA. Similarly, the bimolecular quenching constant *k*_q_ reflects the level of structure in the RNA. Absence of structure would result in a similar level of solvent exposure of 2-AP at all the locations of the RNA. Thus when the RNA is bound to ribosome without preserving its folded structure, we expect the fluorescence lifetime and *k*_q_ of 2-AP to become largely position independent as observed. In contrast, rotational dynamics of 2-AP need not be uniform across the length of ribosome-bound RNA as the strength of binding of the single-stranded RNA is likely to be discernibly position dependent, thus leading to variation in the level of segmental dynamics of the bound RNA which contributes to the mean rotational correlation time of 2-AP. Furthermore, we note that the extent of non-flatness is higher for rotational correlation time when compared to the other two observables even at 45ºC and urea (Figure 3C) where we expect a total loss of secondary structure. The observed dispersion in this case is likely to be caused by site-specific near neighbor interaction.

**Table 2.** Parameters associated with decay of fluorescence intensity, fluorescence anisotropy and bimolecular quenching constant (*k*_q_) of 2-AP in MiniROSE RNA bound to ribosome

2-AP-site^a^	Mean fluorescence lifetime, *τ*_m_(ns)		Mean rotational correlation time^b^, *ϕ*_m_(ns)		*k*_q_^c^ (10^9^ M^−1^s^−1^)	
	20°C	45°C	20°C	45°C	20°C	45°C
6	1.36 ± 0.04	1.04 ± 0.03	28.5 ± 1.5	15.6 ± 0.9	3.8 ± 0.2	5.2 ± 0.3
10	2.00 ± 0.07	1.88 ± 0.08	28.8 ± 1.1	14.8 ± 1.0	2.7 ± 0.1	3.3 ± 0.2
24	1.03 ± 0.05	1.66 ± 0.04	31.4 ± 1.3	9.7 ± 0.8	4.4 ± 0.2	5.9 ± 0.5
27	1.10 ± 0.05	1.53 ± 0.05	24.3 ± 0.9	13.7 ± 1.1	8.9 ± 0.3	4.8 ± 0.2
35	1.31 ± 0.10	1.47 ± 0.05	19.0 ± 1.3	15.3 ± 0.6	5.7 ± 0.2	4.2 ± 0.3
38	1.95 ± 0.03	1.72 ± 0.03	15.6 ± 1.4	22.8 ± 1.3	3.1 ± 0.2	5.9 ± 0.4
41	1.75 ± 0.06	1.65 ± 0.04	25.4 ± 1.2	8.7 ± 0.3	3.0 ± 0.1	4.6 ± 0.3

^a^The number refers to the position of 2-AP in MiniROSE RNA from the 5′-end.

^b^The longer correlation time was arbitrarily taken as 50 ns while estimating *ϕ*_m_( = *β*_1_*ϕ*_1_ + *β*_2_*ϕ*_2_; see Equation (6)).

^c^*k*_q_ is the bimolecular quenching constant determined using the Stern–Volmer equation (Equation (7)).

The loss of folded structure of ribosome-bound MiniROSE RNA at 45°C is best illustrated by the change in fluorescence lifetime of 2-AP located at the SD region. As mentioned above, a shorter value of mean fluorescence lifetime of 2-AP is indicative of base pairing and hence the presence of folded structure of MiniROSE RNA (21–23,26). Significant increase in the value of mean lifetime for 2-AP at positions 24 and 27 of ribosome-bound MiniROSE RNA when the temperature was raised from 20 to 45°C (Table 2) to values seen in the presence of urea at 45°C (Table 1) is a strong indicator of melting of structure of ribosome-bound MiniROSE RNA at 45°C. Loss of folded structure ‘at the SD sequence’ leads to productive binding to ribosome resulting in initiation of translation. Furthermore, it is interesting to note that fluorescence lifetime of 2-AP at positions 24 and 27 did not change appreciably between 20 and 45°C in the absence of ribosome (Table 1), indicating the role played by ribosome in releasing the SD sequence. The dispersion in quenching constant reduces at 20°C when MiniROSE RNA binds ribosome (compare Figures 2 and 5), which reduces further at 45°C, again underscoring the importance of ribosome binding in RNA structural transition.

### Mechanism of action of the RNA thermometer

**Figure 7.**
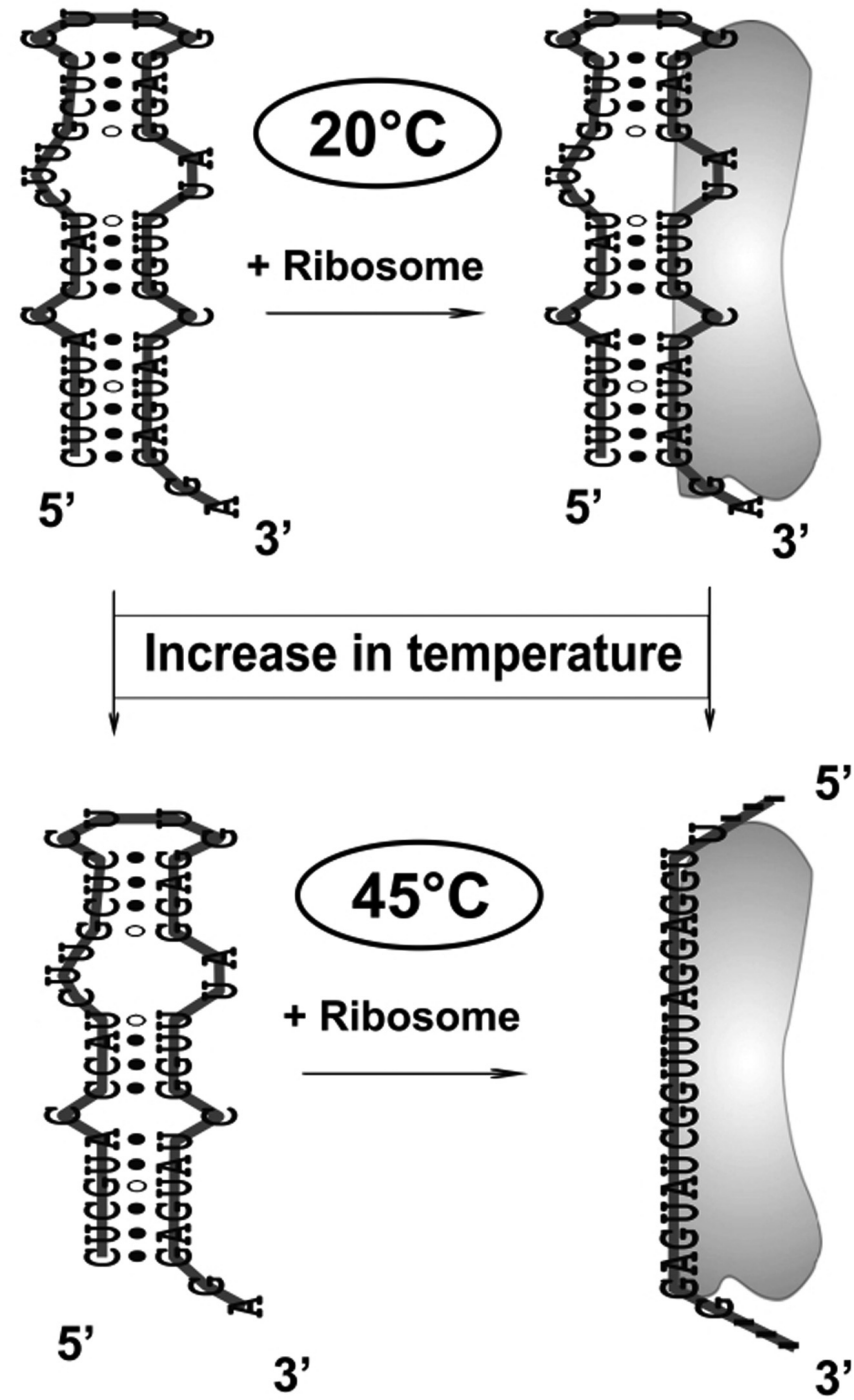
The scheme illustrating the mechanism of temperature-induced structural change in ribosome-bound MiniROSE RNA resulting in inhibition of translation at 20ºC and its promotion at 45ºC following the release of the SD sequence for productive binding to ribosome. Increase in the level of dynamics of the free RNA at 45°C is indicated as weakening of base pairs.

The results presented in the current study tend to provide a novel mechanism for temperature control of translation provided by the MiniROSE RNA thermometer. According to our model (Figure 7), the MiniROSE segment of RNA binds to ribosome even at temperatures as low as 20°C; however, its folded structure, especially at the SD sequence region, remains preserved even while bound to ribosome at this temperature resulting in retardation of translation. However, higher temperatures such as 45°C cause the ribosome-bound-folded structure of RNA to open up liberating the SD sequence to bind to ribosome in a manner that is proficient in triggering translation initiation. Thus, the ribosome surface plays an important role in promoting the local melting of essential regions of RNA at moderately high temperature of 45°C. The mechanics of ribosome surface in assisting RNA unfolding specifically at higher temperature might constitute an essential part of thermometer function. In the absence of ribosomes, transition to high temperature simply enhances the local dynamics of bases, perhaps involving no large-scale opening-up of the base-paired structure, which in turn may not be biologically very relevant.

Thus, this model differs significantly from models based on temperature-dependent (20–45°C) destabilization of the RNA structural motif and subsequent binding of the SD region of the RNA to ribosome (9). Observation of slow rotational correlation time constant >50 ns in the presence of ribosome at both 20 and 45°C (Figure 4) is an unequivocal evidence for binding of the RNA at both the temperatures. Obviously, the mode of binding to ribosome is quite different at these two temperatures, which is critical in promoting RNA melting congenial for translation, the details of which hold the mechanics of thermometer function.

That the folded structure of MiniROSE RNA is largely intact at 45°C is seen from site-specific dispersion (non-flatness) of fluorescence parameters associated with 2-AP (Figure 3). Loss of folded structure is demonstrated by near-flat profile of the parameters seen at 45°C in the presence of urea which is expected to abrogate intramolecular interactions. In fact, global melting of MiniROSE RNA, monitored by absorbance at 260 nm, occurs in the temperature region of 60–70°C, with little changes occurring in the region of 20–45°C (data not shown) similar to earlier observations (9). Measurement of global melting in the presence of ribosomes is impractical due to high absorbance from ribosomes at 260 nm. Furthermore, local melting at the SD region is more relevant to the mechanism of translational control when compared to the overall melting of the hairpin structure. Although destabilization of the RNA structure (in the absence of ribosome) local to the region of SD sequence at 42°C was inferred from NMR studies, these studies were carried out with MicroROSE RNA (a smaller version of MiniROSE RNA, (9)), which had an engineered loop (CUUG changed to UUCG), an artificial sequence by design. In contrast, our samples had the native loop sequence, which is more relevant in revealing the mechanism of thermometer action. Alternatively, the enhanced dynamics observed at all the locations of MiniROSE RNA at 45°C when compared to that at 20°C (Rotational correlation time in Table 1) in the absence of ribosome could be the channel through which the ‘open’ form of RNA would bind to ribosome in a productive manner. The enhanced dynamics at 45°C (in the absence of ribosome) could have resulted in the absence of imino-proton resonances corresponding to the SD region leading to the conclusion that this segment is largely open at 45°C (9). It is fortuitously possible that the engineered hairpin loop used in earlier studies structurally mimics some features of ribosome-bound form of natural MiniROSE RNA (used in the current study), which might have resulted in high levels of temperature-caused transitions observed earlier, even in the absence of ribosomes. This could also be seen in the temperature dependence of NMR spectra (Figure 2). It is therefore relevant to address these issues by comparing both types of MiniROSE RNA (engineered versus natural) side-by-side carefully in future studies.

Temperature sensing by the ROSE RNA sequence requires the presence of G15, a highly conserved nucleotide (9,49). Deletion of G15 results in abolition of translation at both low and high temperatures. We measured fluorescence lifetimes of 2-AP at positions 24, 27 and 35 in the MiniROSE RNA, wherein G15 was deleted. The results are shown in Table 3 and Supplementary Table S5. We note that the mean lifetime *τ*_m_ did not increase appreciably (indicating opening of the hairpin structure) when the temperature was increased from 20°C to 45°C especially in the presence of ribosome (Table 3 and Supplementary Table S5) in contrast to observations with the wild-type MiniROSE RNA (Table 2). It is worth mentioning here again that the RNA binding to ribosome was confirmed by the emergence of a long (>50 ns) rotational correlation time as mentioned earlier (data not shown). These results show that the SD sequence of 15D-ROSE RNA is not liberated at 45°C for ‘productive’ binding to ribosome, a conclusion that offers additional support to our hypothesis of ribosome-binding-induced liberation of SD sequence at 45°C for the wild-type MiniROSE RNA. Thus, binding occurs without opening of the SD sequence, which perhaps models the temperature-insensitive state of binding, only following which the melting of SD sequence ensues. Unraveling finer details of mechanistic steps requires a study that encompasses active components of translational initiation in addition to that of ribosomal components in the reaction system.

**Table 3.** Parameters associated with decay of fluorescence intensities of 2-AP in MiniROSE RNA when G at 15 position was deleted

2-AP-site^a^	Mean fluorescence lifetimes, *τ*_m_(ns)				
	20°C		45°C		45°C + urea
	Without ribosome	With ribosome	Without ribosome	With ribosome	Without ribosome
24MR15D	0.38 ± 0.02	0.87 ± 0.04	0.48 ± 0.03	0.57 ± 0.02	1.41 ± 0.05
27MR15D	0.31 ± 0.02	0.65 ± 0.03	0.39 ± 0.02	0.75 ± 0.04	1.12 ± 0.04
35MR15D	0.30 ± 0.01	0.47 ± 0.03	0.28 ± 0.01	0.41 ± 0.02	1.09 ± 0.04

^a^The number refers to the position of 2-AP in MiniROSE RNA from the 5′-end. 15D denotes the deletion of Guanine from position 15.

The scheme shown in Figure 7 describes a minimal model invoking four states of the MiniROSE RNA thermometer, as studied in the current investigation. One could visualize a more complex scheme such as partially folded RNA structure both in the free and ribosome-bound forms. This would require more information on structure and dynamics to a higher level of precision. Such a scheme of enhanced complexity has been recently proposed in an adenine-sensing riboswitch (8). The important take-home in the model described here is the last state where the MiniROSE RNA thermometer is unfolded at higher temperature, only when it is ribosome bound. We hope to delineate the details of ribosome interaction that facilitates RNA-thermometer sequence unfolding at higher temperature in our future studies. We believe that SD-sequence accessibility for ribosome recognition is coupled to ribosome function, only assisted by sensing at higher temperature, which is classically referred as thermometer function. Although the present work reveals the need of binding to ribosome for the thermometer function of the MiniROSE RNA, it would be appropriate to point out that the need of ribosome binding may not be a general requirement as shown by temperature-induced opening of the 4U RNA thermometer in the absence of ribosome (15,16). This point is relevant in lieu of the limited number of mechanistic studies on temperature-sensing riboswitches (3).

Finally, this work demonstrates the use of site-specific fluorescence dynamics as an effective tool in unraveling molecular mechanisms of RNA structures. Fluorescence-based techniques score over high-resolution structural methods such as NMR by their ability to provide residue-specific information on structure and dynamics of macromolecular systems with little restriction on their size and complexity of interaction systems, such as translation machines (ribosomes), etc. Our study strongly underscores the need to investigate RNA switches (thermometers/riboswitches, etc.) in their native interaction milieu also apart from studying them as free RNA molecules.

## SUPPLEMENTARY DATA

Supplementary Data are available at NAR Online.

SUPPLEMENTARY DATA
