# Supplementary material for: Site-specific fluorescence dynamics in an RNA ‘thermometer’ reveals the role of ribosome binding in its temperature-sensitive switch function
Source: Nucleic Acids Res. 2014 Dec 3;43(1):493–503. doi: 10.1093/nar/gku1264 (PMC4288164; doi:10.1093/nar/gku1264)
Supplement: SUPPLEMENTARY DATA [file supp_43_1_493__index.html]

Site-specific fluorescence dynamics in an RNA ‘thermometer’ reveals the role of ribosome binding in its temperature-sensitive switch function — Site-specific fluorescence dynamics in an RNA ‘thermometer’ reveals the role of ribosome binding in its temperature-sensitive switch function — Site-specific fluorescence dynamics in an RNA ‘thermometer’ reveals the role of ribosome binding in its temperature-sensitive switch function — SUPPLEMENTARY DATA 

# Site-specific fluorescence dynamics in an RNA ‘thermometer’ reveals the role of ribosome binding in its temperature-sensitive switch function

## SUPPLEMENTARY DATA

**Files in this Data Supplement:**

- SUPPLEMENTARY DATA
