# Supplementary material for: Site-specific fluorescence dynamics in an RNA ‘thermometer’ reveals the role of ribosome binding in its temperature-sensitive switch function
Source: Nucleic Acids Res. 2014 Dec 3;43(1):493–503. doi: 10.1093/nar/gku1264 (PMC4288164; doi:10.1093/nar/gku1264)
Supplement: SUPPLEMENTARY DATA [file supp_gku1264_nar-02478-f-2014-File009.docx]

**Table S1. Parameters associated with fluorescence intensity decay of 2-AP in MiniROSE RNA**

| 2-AP-site^a^ | Fluorescence lifetime, τ (ns) and amplitude, α | | | | | | | | | | | | | | |
| --- | --- | --- | --- | --- | --- | --- | --- | --- | --- | --- | --- | --- | --- | --- | --- |
|  | 20°C | | | | | 45°C | | | | | 45°C with urea | | | | |
|  | τ_1_  α_1_ | τ_2_  α_2_ | τ_3_  α_3_ | τ_4_  α_4_ | τ_m_ | τ_1_  α_1_ | τ_2_  α_2_ | τ_3_  α_3_ | τ_4_  α_4_ | τ_m_ | τ_1_  α_1_ | τ_2_  α_2_ | τ_3_  α_3_ | τ_4_  α_4_ | τ_m_ |
| 6 | 0.13  ±0.03  0.30  ±0.04 | 0.59  ±0.04  0.50  ±0.01 | 1.83  ±0.19  0.13  ±0.02 | 7.28  ±0.33  0.07  ±0.01 | 1.06  ±0.05 | 0.08  ±0.03  0.38  ±0.05 | 0.37  ±0.03  0.47  ±0.05 | 1.49  ±0.14  0.10  ±0.01 | 5.12  ±0.24  0.05  ±0.01 | 0.60  ±0.06 | 0.22  ±0.02 0.50  ±0.04 | 1.11  ±0.07 0.28  ±0.02 | 4.02  ±0.16 0.22  ±0.03 |  | 1.30  ±0.04 |
| 10 | 0.09  ±0.01  0.40  ±0.01 | 0.55  ±0.01  0.28  ±0.01 | 3.43  ±0.04  0.20  ±0.01 | 7.95  ±0.13  0.12  ±0.01 | 1.86  ±0.05 | 0.22  ±0.01  0.30  ±0.02 | 0.84  ±0.08  0.25  ±0.02 | 2.77  ±0.16  0.35  ±0.02 | 6.51  ±0.28  0.10  ±0.01 | 1.88  ±0.04 | 0.06  ±0.01  0.35  ±0.02 | 0.44  ±0.03  0.15  ±0.01 | 1.88  ±0.15 0.22  ±0.02 | 4.18  ±0.25 0.28  ±0.03 | 1.65  ±0.05 |
| 24 | 0.11  ±0.02  0.57  ±0.02 | 0.67  ±0.13  0.18  ±0.01 | 3.26  ±0.37  0.13  ±0.01 | 9.20  ±0.29  0.12  ±0.01 | 1.69  ±0.08 | 0.20  ±0.02  0.43  ±0.02 | 0.83  ±0.09  0.19  ±0.01 | 2.68  ±0.17  0.20  ±0.01 | 7.34  ±0.14  0.18  ±0.01 | 2.12  ±0.01 | 0.14  ±0.02 0.40  ±0.03 | 0.94  ±0.09  0.29  ±0.02 | 2.96  ±0.13 0.21  ±0.01 | 7.25  ±0.18  0.10  ±0.01 | 1.67  ±0.03 |
| 27 | 0.09  ±0.01  0.59  ±0.02 | 0.67  ±0.05  0.15  ±0.01 | 3.12  ±0.09  0.19  ±0.01 | 8.05  ±0.27  0.07  ±0.01 | 1.34  ±0.06 | 0.14  ±0.01  0.47  ±0.01 | 0.87  ±0.08  0.20  ±0.02 | 2.47  ±0.10  0.26  ±0.02 | 6.49  ±0.22  0.07  ±0.01 | 1.32  ±0.02 | 0.20  ±0.02 0.29  ±0.01 | 1.02  ±0.06 0.26  ±0.02 | 2.85  ±0.13 0.41  ±0.03 | 6.07  ±0.25 0.04  ±0.01 | 1.72  ±0.06 |
| 35 | 0.12  ±0.01  0.32  ±0.02 | 0.57  ±0.05  0.29  ±0.01 | 2.46  ±0.02  0.24  ±0.01 | 7.17  ±0.19  0.15  ±0.01 | 1.82  ±0.01 | 0.17  ±0.01  0.32  ±0.01 | 0.67  ±0.05  0.31  ±0.01 | 2.36  ±0.07  0.28  ±0.01 | 6.14  ±0.13  0.09  ±0.01 | 1.49  ±0.02 | 0.52  ±0.04 0.32  ±0.03 | 2.15  ±0.06 0.39  ±0.02 | 4.24  ±0.13  0.29  ±0.02 |  | 2.22  ±0.07 |
| 38 | 0.09  ±0.01  0.69  ±0.01 | 0.65  ±0.04  0.17  ±0.01 | 2.90  ±0.13  0.09  ±0.01 | 9.75  ±0.12  0.05  ±0.01 | 0.91  ±0.05 | 0.08  ±0.01  0.57  ±0.02 | 0.54  ±0.01  0.17  ±0.01 | 2.19  ±0.14  0.19  ±0.01 | 6.66  ±0.37  0.07  ±0.01 | 1.01  ±0.03 | 0.30  ±0.02 0.34  ±0.03 | 1.57  ±0.13  0.34  ±0.02 | 3.96  ±0.21 0.32  ±0.03 |  | 1.90  ±0.03 |
| 41 | 0.05  ±0.01  0.67  ±0.01 | 0.68  ±0.08  0.09  ±0.01 | 3.82  ±0.16  0.16  ±0.01 | 7.02  ±0.22  0.08  ±0.01 | 1.22  ±0.05 | 0.04  ±0.01  0.56  ±0.04 | 0.43  ±0.04  0.12  ±0.01 | 2.55  ±0.12  0.24  ±0.01 | 5.1  ±0.36  0.08  ±0.02 | 1.05  ±0.09 | 0.30  ±0.01  0.34  ±0.02 | 1.89  ±0.05 0.33  ±0.03 | 4.56  ±0.31 0.33  ±0.01 |  | 2.22  ±0.05 |
| ^a^The number refers to the position of 2-AP in MiniROSE RNA from the 5’-end. | | | | | | | | | | | | | | | |

**Table S2. Parameters associated with decay of fluorescence anisotropy of 2-AP in MiniROSE RNA**

| 2-AP-site^a^ | Rotational correlation time, ϕ, ns  Amplitude, β | | | | | |
| --- | --- | --- | --- | --- | --- | --- |
|  | 20°C | | 45°C | | 45°C + Urea | |
|  | ϕ_1_  β_1_ | ϕ_2_  β_2_ | ϕ_1_  β_1_ | ϕ_2_  β_2_ | ϕ_1_  β_1_ | ϕ_2_  β_2_ |
| 6 | 2.50  ±0.18  0.11  ±0.03 | 2.90  ±0.23  0.89  ±0.05 | 0.71  ±0.06  0.75  ±0.04 | 3.55  ±0.13  0.25  ±0.02 | 0.36  ±0.02  0.54  ±0.03 | 1.72  ±0.09  0.46  ±0.04 |
| 10 | 0.70  ±0.04  0.63  ±0.04 | 8.15  ±0.43  0.37  ±0.02 | 0.27  ±0.03  0.65  ±0.02 | 2.03  ±0.07  0.35  ±0.03 | 0.38  ±0.05  0.60  ±0.05 | 1.53  ±0.10  0.40  ±0.02 |
| 24 | 0.40  ±0.02  0.61  ±0.04 | 6.70  ±0.38  0.39  ±0.03 | 0.17  ±0.01  0.63  ±0.02 | 1.90  ±0.08  0.37  ±0.04 | 0.23  ±0.02  0.48  ±0.05 | 1.07  ±0.04  0.52  ±0.03 |
| 27 | 0.33  ±0.04  0.67  ±0.06 | 4.02  ±0.21  0.33  ±0.02 | 0.18  ±0.02  0.75  ±0.04 | 1.06  ±0.09  0.25  ±0.01 | 0.27  ±0.03  0.66  ±0.03 | 1.52  ±0.06  0.34  ±0.04 |
| 35 | 0.55  ±0.04  0.74  ±0.02 | 4.50  ±0.31  0.26  ±0.03 | 0.36  ±0.03  0.76  ±0.05 | 4.15  ±0.23  0.24  ±0.02 | 0.37  ±0.04  0.73  ±0.03 | 1.40  ±0.10  0.27  ±0.01 |
| 38 | 0.25  ±0.02  0.56  ±0.05 | 3.88  ±0.15  0.44  ±0.03 | 0.16  ±0.01  0.68  ±0.04 | 1.27  ±0.08  0.32  ±0.03 | 0.36  ±0.02  0.55  ±0.02 | 1.82  ±0.09  0.45  ±0.01 |
| 41 | 0.33  ±0.03  0.73  ±0.04 | 3.13  ±0.13  0.27  ±0.02 | 0.04  ±0.01  0.36  ±0.02 | 0.51  ±0.02  0.64  ±0.03 | 0.31  ±0.04  0.74  ±0.05 | 1.51  ±0.08  0.26  ±0.02 |
| ^a^The number refers to the position of 2-AP in MiniROSE RNA from the 5’-end. | | | | | | |

**Table S3. Parameters associated with decay of fluorescence intensity of 2-AP in MiniROSE RNA bound to ribosome**

| 2-AP-site^a^ | Fluorescence lifetime, τ(ns) and amplitude, α | | | | | | | | | |
| --- | --- | --- | --- | --- | --- | --- | --- | --- | --- | --- |
|  | 20^0^C | | | | | 45^0^C | | | | |
|  | τ_1_  α_1_ | τ_2_  α_2_ | τ_3_  α_3_ | τ_4_  α_4_ | τ_m_ | τ_1_  α_1_ | τ_2_  α_2_ | τ_3_  α_3_ | τ_4_  α_4_ | τ_m_ |
| 6 | 0.07  ±0.01  0.34  ±0.03 | 0.55  ±0.05  0.40  ±0.01 | 2.36  ±0.16  0.17  ±0.02 | 7.67  ±0.12  0.09  ±0.01 | 1.36  ±0.04 | 0.12  ±0.01  0.36  ±0.04 | 0.47  ±0.03  0.42  ±0.03 | 2.15  ±0.08  0.15  ±0.01 | 6.29  ±0.28  0.07  ±0.01 | 1.04  ±0.03 |
| 10 | 0.07  ±0.01  0.46  ±0.02 | 0.62  ±0.08  0.20  ±0.01 | 3.23  ±0.35  0.19  ±0.02 | 8.53  ±0.66  0.15  ±0.02 | 2.00  ±0.07 | 0.13  ±0.01  0.35  ±0.03 | 0.85  ±0.05  0.24  ±0.02 | 2.96  ±0.12  0.31  ±0.02 | 6.97  ±0.26  0.10  ±0.01 | 1.88  ±0.08 |
| 24 | 0.06  ±0.01  0.62  ±0.02 | 0.55  ±0.06  0.17  ±0.01 | 2.47  ±0.25  0.13  ±0.01 | 7.28  ±0.34  0.08  ±0.01 | 1.03  ±0.05 | 0.10  ±0.01  0.43  ±0.02 | 0.66  ±0.05  0.23  ±0.01 | 2.46  ±0.13  0.23  ±0.01 | 7.81  ±0.16  0.12  ±0.01 | 1.66  ±0.04 |
| 27 | 0.04  ±0.01  0.61  ±0.03 | 0.45  ±0.06  0.15  ±0.01 | 2.47  ±0.17  0.15  ±0.01 | 6.79  ±0.09  0.09  ±0.01 | 1.10  ±0.05 | 0.11  ±0.01  0.37  ±0.02 | 0.63  ±0.04  0.23  ±0.01 | 2.37  ±0.08  0.31  ±0.01 | 6.67  ±0.15  0.09  ±0.01 | 1.53  ±0.05 |
| 35 | 0.04  ±0.01  0.52  ±0.02 | 0.45  ±0.04  0.19  ±0.01 | 2.36  ±0.04  0.18  ±0.01 | 7.01  ±0.12  0.11  ±0.01 | 1.31  ±0.10 | 0.12  ±0.01  0.38  ±0.02 | 0.71  ±0.06  0.27  ±0.01 | 2.53  ±0.22  0.26  ±0.01 | 6.13  ±0.41  0.09  ±0.01 | 1.47  ±0.05 |
| 38 | 0.11  ±0.01  0.37  ±0.02 | 0.65  ±0.08  0.26  ±0.02 | 2.84  ±0.26  0.22  ±0.01 | 7.51  ±0.23  0.15  ±0.02 | 1.95  ±0.03 | 0.15  ±0.01  0.37  ±0.03 | 0.69  ±0.04  0.26  ±0.02 | 2.50  ±0.09  0.25  ±0.01 | 7.14  ±0.14  0.12  ±0.01 | 1.72  ±0.03 |
| 41 | 0.08  ±0.01  0.45  ±0.03 | 0.66  ±0.07  0.21  ±0.01 | 3.30  ±0.17  0.21  ±0.01 | 7.14  ±0.09  0.12  ±0.01 | 1.75  ±0.06 | 0.09  ±0.02  0.38  ±0.01 | 0.69  ±0.07  0.19  ±0.01 | 2.85  ±0.06  0.36  ±0.01 | 6.14  ±0.25  0.07  ±0.01 | 1.65  ±0.04 |
| ^a^The number refers to the position of 2-AP in MiniROSE RNA from the 5’-end. | | | | | | | | | | |

**Table S4. Parameters associated with decay of fluorescence anisotropy and bimolecular quenching constant (k_q_)of 2-AP in MiniROSE RNA bound to ribosome**

| 2-AP-site^a^ | Rotational correlation time, ϕ(ns) Amplitude, β | | | |
| --- | --- | --- | --- | --- |
|  | 20°C | | 45°C | |
|  | ϕ_1_  β_1_ | ϕ_2_  β_2_ | ϕ_1_  β_1_ | ϕ_2_  β_2_ |
| 6 | 1.05  ±0.04  0.44  ±0.03 | >50.0  0.56  ±0.04 | 0.82  ±0.03  0.70  ±0.02 | >50.0  0.30  ±0.03 |
| 10 | 0.73  ±0.05  0.43  ±0.03 | >50.0  0.57  ±0.04 | 0.53  ±0.02  0.71  ±0.04 | >50.0  0.29  ±0.02 |
| 24 | 1.02  ±0.04  0.38  ±0.03 | >50.0  0.62  ±0.03 | 0.27  ±0.01  0.81  ±0.04 | >50.0  0.19  ±0.02 |
| 27 | 0.54  ±0.03  0.52  ±0.02 | >50.0  0.48  ±0.03 | 0.35  ±0.03  0.72  ±0.04 | >50.0  0.28  ±0.02 |
| 35 | 0.82  ±0.05  0.63  ±0.03 | >50.0  0.37  ±0.02 | 0.45  ±0.02  0.69  ±0.04 | >50.0  0.31  ±0.03 |
| 38 | 0.95  ±0.06  0.70  ±0.04 | >50.0  0.30  ±0.02 | 0.46  ±0.04  0.55  ±0.02 | >50.0  0.45  ±0.04 |
| 41 | 0.76  ±0.05  0.50  ±0.04 | >50.0  0.50  ±0.04 | 0.26  ±0.01  0.83  ±0.03 | >50.0  0.17  ±0.01 |
| ^a^The number refers to the position of 2-AP in MiniROSE RNA from the 5’-end. The low limit of the longer correlation time (>50ns) is due to the finite limit of the time window of observation set by the fluorescence lifetime. | | | | |

**Table S5. Parameters associated with decay of fluorescence intensity of 2-AP in MiniROSE RNA when G at 15 position was deleted.**

| 2-AP-site^a^ | | Fluorescence lifetime, τ(ns) and amplitude, α | | | | | | | | | |
| --- | --- | --- | --- | --- | --- | --- | --- | --- | --- | --- | --- |
|  |  | Without ribosome | | | | | With ribosome | | | | |
|  |  | τ_1_  α_1_ | τ_2_  α_2_ | τ_3_  α_3_ | τ_4_  α_4_ | τ_m_ | τ_1_  α_1_ | τ_2_  α_2_ | τ_3_  α_3_ | τ_4_  α_4_ | τ_m_ |
| 20^0^C | 24MR15D | 0.03  ±0.00  0.84  ±0.03 | 0.44  ±0.04  0.09  ±0.01 | 3.04  ±0.06  0.05  ±0.01 | 9.13  ±0.10  0.02  ±0.00 | 0.38  ±0.02 | 0.07  ±0.01  0.63  ±0.05 | 0.67  ±0.07  0.19  ±0.02 | 2.91  ±0.13  0.14  ±0.01 | 7.22  ±0.21  0.04  ±0.00 | 0.87  ±0.04 |
|  | 27MR15D | 0.03  ±0.00  0.87  ±0.04 | 0.42  ±0.03  0.08  ±0.00 | 2.57  ±0.07  0.03  ±0.00 | 7.20  ±0.10  0.02  ±0.00 | 0.31  ±0.02 | 0.04  ±0.00  0.71  ±0.03 | 0.53  ±0.05  0.14  ±0.01 | 2.68  ±0.12  0.11  ±0.01 | 6.77  ±0.21  0.04  ±0.00 | 0.65  ±0.03 |
|  | 35MR15D | 0.04  ±0.00  0.84  ±0.05 | 0.37  ±0.02  0.09  ±0.01 | 2.17  ±0.06  0.05  ±0.01 | 6.39  ±0.11  0.02  ±0.00 | 0.30  ±0.01 | 0.04  ±0.01  0.78  ±0.06 | 0.46  ±0.02  0.12  ±0.01 | 2.60  ±0.09  0.08  ±0.01 | 6.78  ±0.20  0.03  ±0.00 | 0.47  ±0.03 |
| 45^0^C | 24MR15D | 0.04  ±0.00  0.79  ±0.03 | 0.48  ±0.04  0.11  ±0.01 | 2.63  ±0.10  0.07  ±0.01 | 7.03  ±0.13  0.03  ±0.00 | 0.48  ±0.03 | 0.04  ±0.00  0.69  ±0.03 | 0.43  ±0.03  0.14  ±0.01 | 1.83  ±0.13  0.11  ±0.01 | 5.08  ±0.16  0.06  ±0.00 | 0.57  ±0.02 |
|  | 27MR15D | 0.04  ±0.00  0.78  ±0.04 | 0.44  ±0.03  0.11  ±0.01 | 2.11  ±0.07  0.08  ±0.01 | 5.66  ±0.09  0.03  ±0.00 | 0.39  ±0.02 | 0.06  ±0.00  0.61  ±0.05 | 0.53  ±0.04  0.19  ±0.02 | 2.42  ±0.14  0.16  ±0.01 | 5.92  ±0.24  0.04  ±0.00 | 0.75  ±0.04 |
|  | 35MR15D | 0.04  ±0.00  0.83  ±0.04 | 0.41  ±0.02  0.09  ±0.02 | 2.08  ±0.10  0.06  ±0.00 | 6.06  ±0.12  0.02  ±0.00 | 0.28  ±0.01 | 0.05  ±0.00  0.76  ±0.04 | 0.36  ±0.02  0.12  ±0.01 | 1.96  ±0.07  0.09  ±0.01 | 5.33  ±0.18  0.03  ±0.00 | 0.41  ±0.02 |
| 45^0^C + Urea | 24MR15D | 0.08  ±0.01  0.49  ±0.04 | 0.96  ±0.06  0.24  ±0.02 | 3.64  ±0.07  0.24  ±0.01 | 8.26  ±0.09  0.03  ±0.00 | 1.41  ±0.05 |  |  |  |  |  |
|  | 27MR15D | 0.08  ±0.01  0.48  ±0.03 | 0.62  ±0.03  0.21  ±0.01 | 2.54  ±0.10  0.27  ±0.02 | 6.40  ±0.11  0.04  ±0.00 | 1.12  ±0.04 |  |  |  |  |  |
|  | 35MR15D | 0.13  ±0.01  0.43  ±0.02 | 0.63  ±0.03  0.19  ±0.01 | 1.99  ±0.06  0.32  ±0.03 | 4.30  ±0.15  0.07  ±0.01 | 1.09  ±0.04 |  |  |  |  |  |
| ^a^The number refers to the position of 2-AP in MiniROSE RNA from the 5’-end. 15 D denotes the deletion of Guanine from position 15. | | | | | | | | | | | |
